# Bevacizumab plus nab‐paclitaxel and platinum as second‐line therapy for driver‐gene‐negative non‐squamous non‐small cell lung cancer after immunochemotherapy: A prospective single‐arm single‐centre study

**DOI:** 10.1002/ctm2.70814

**Published:** 2026-09-11

**Authors:** Ying Lin, Yunbo Yu, Zhihuang Hu, Chenchen Wei, Zezhou Wang, Yao Zhang, Zhenhua Wu, Bo Yu, Xinmin Zhao, Hui Yu, Xianghua Wu, Huijie Wang, Jialei Wang

**Affiliations:** ^1^ Department of Medical Oncology Fudan University Shanghai Cancer Centre Shanghai China; ^2^ Department of Oncology Shanghai Medical College Fudan University Shanghai China; ^3^ Department of Oncology The Second Affiliated Hospital of Nanjing Medical University Nanjing China; ^4^ Department of Cancer Prevention Fudan University Shanghai Cancer Centre Shanghai China; ^5^ Shanghai Municipal Hospital Oncological Specialist Alliance Shanghai China; ^6^ Institute of Thoracic Oncology Fudan University Shanghai China

**Keywords:** bevacizumab, nab‐paclitaxel, non‐squamous non‐small cell lung cancer, platinum re‐challenge, second‐line therapy, TWEAK

## Abstract

**Background:**

Effective second‐line therapy is urgently needed for patients with driver‐gene‐negative non‐squamous non‐small cell lung cancer (nsqNSCLC) who progress after first‐line immunochemotherapy. This study aims to evaluate the efficacy and safety of bevacizumab combined with nab‐paclitaxel and platinum (the BAP regimen) in this setting.

**Methods:**

This prospective, single‐arm, single‐centre study enrolled 56 patients with advanced driver‐gene‐negative nsqNSCLC failing first‐line immunochemotherapy. Patients received bevacizumab, nab‐paclitaxel, and platinum (carboplatin or cisplatin) for 4–6 cycles, followed by bevacizumab maintenance. The primary endpoint was objective response rate (ORR). Secondary endpoints included progression‐free survival (PFS), overall survival (OS), disease control rate (DCR) and safety.

**Results:**

All patients had failed first‐line platinum‐based immunochemotherapy. The confirmed ORR was 46.4% (26/56, 95% confidence interval [CI]: 34.0%–59.3%, *p* < 0.001), and DCR was 75%. The median PFS and OS were 5.6 (95% CI: 4.35–6.79) and 18.8 (95% CI: 10.31–27.24) months. Patients with ≤ 2 metastatic organs had a significantly longer median PFS than those with >2 metastatic organs (*p* = 0.014). Multivariable Cox analysis showed that a platinum‐free interval <6 months was an independent biomarker of worse PFS. The safety profile was manageable. Plasma proteomics identified baseline high TWEAK level as a potential biomarker for response to both the second‐line BAP regimen and first‐line immunochemotherapy. Patients with high baseline TWEAK levels before first‐line immunochemotherapy showed a median OS of 39.8 months under this sequential treatment modality.

**Conclusions:**

The BAP regimen demonstrated preliminary efficacy with manageable safety as a second‐line therapy for patients with advanced driver‐gene‐negative nsqNSCLC after immunochemotherapy. Plasma TWEAK levels may serve as a potential biomarker to help identify patients who might benefit most from this sequential treatment modality, pending further independent validation. Further studies are needed.

## Graphical abstract

This study prospectively evaluated the efficacy and safety of the bevacizumab, nab‐paclitaxel and platinum combination as a second‐line therapy for driver‐gene‐negative advanced non‐squamous non‐small cell lung cancer after immunochemotherapy. The confirmed objective response rate was 46.4%. The median progression‐free survival and overall survival were 5.6 and 18.8 months. The toxicity was manageable.

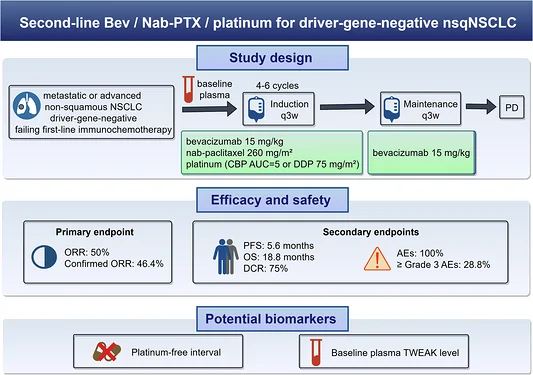

**LIST OF ABBREVIATIONS**
ADC: antibody‐drug conjugate
AE: adverse event
CI: confidence interval
CR: complete response
CT: computed tomography
DCR: disease control rate
DpR: depth of response
FDR: false discovery rate
ICI: immune checkpoint inhibitor
ITT: intent‐to‐treat
NE: not evaluable
NSCLC: non‐small cell lung cancer
nsqNSCLC: non‐squamous non‐small cell lung cancer
ORR: objective response rate
OS: overall survival
PFI: platinum‐free interval
PFS: progression‐free survival
PP: per‐protocol
PR: partial response
RECIST: Response Evaluation Criteria in Solid Tumours
VEGF: vascular endothelial growth factor

## 1 INTRODUCTION

Lung cancer remains one of the most prevalent and lethal malignancies worldwide.^1^ Immune checkpoint inhibitors (ICIs) have revolutionised the treatment landscape for non‐small cell lung cancer (NSCLC), especially for patients with driver‐gene‐negative NSCLC.^2^ PD‐1 or PD‐L1 inhibitors are the most widely used ICIs. Several Phase III trials have demonstrated that adding a PD‐1/PD‐L1 inhibitor to platinum‐based chemotherapy significantly prolonged progression‐free survival (PFS) and overall survival (OS) in driver‐gene‐negative advanced non‐squamous NSCLC (nsqNSCLC).^2^ Unlike ICI monotherapy, which requires certain PD‐L1 expression thresholds, ICI and chemotherapy combinations confer survival benefits regardless of PD‐L1 status.^2^ Another approach is to incorporate an antiangiogenic strategy into immunochemotherapy, as demonstrated in the IMpower150 trial.^3^ However, the large randomised phase III APPLE trial failed to demonstrate benefit from adding bevacizumab to platinum‐doublet chemotherapy plus atezolizumab in metastatic or recurrent nsqNSCLC.^4^ Overall, ICIs plus pemetrexed and platinum have become the cornerstone for first‐line treatment of driver‐gene‐negative nsqNSCLC.^2^ Despite these advances, most patients ultimately experience disease progression and resistance to immunotherapy.^5^ Current second‐line options include docetaxel, used as a single agent or in combination with antiangiogenic agents such as ramucirumab or nintedanib.^5^ However, the prognosis of patients who progress after immunotherapy remains poor, posing a significant clinical challenge.

New post‐immunotherapy strategies are under intense investigation, but no new standard has yet been established. One approach is to continue ICIs beyond progression and add targeted drugs. In the HUDSON study, the combination of durvalumab and ceralasertib improved outcomes in patients with *ATM* alterations.^6^ In the Lung‐MAP S1800A trial, pembrolizumab plus ramucirumab significantly improved outcomes versus docetaxel or physicians’ choice in ICI‐pretreated NSCLC.^7^ Both combinations are currently under further evaluation (NCT03334617 and NCT05633602, respectively). However, other ICI‐based combinations, including pembrolizumab plus lenvatinib (LEAP‐008 trial), nivolumab plus sitravatinib (SAPPHIRE trial) and atezolizumab plus cabozantinib (CONTACT‐01 trial), failed to show significant survival benefits over docetaxel in large clinical trials.^8^, ^9^, ^10^ In addition, the objective response rates (ORRs) of these regimens were modest, ranging from 11.8% to 22.7%. Antibody‐drug conjugates (ADCs) represent another emerging strategy. Although promising in other tumours, two TROP‐2 ADCs failed to show survival benefits in ICI‐pretreated nsqNSCLC in the TROPION‐Lung01 and EVOKE‐01 trials.^11^, ^12^ Other modalities, including CEACAM5 ADC, bispecific antibodies and cancer vaccines, are also being explored.^5^ Although these agents offer new approaches, most remain in early phases, with efficacy and safety yet to be confirmed.

Antiangiogenesis is an important anticancer strategy and has gained prominence in the era of immunotherapy. By inhibiting vascular endothelial growth factor (VEGF) signalling, antiangiogenic agents suppress tumour angiogenesis and remodel the tumour microenvironment, exhibiting synergy with chemotherapy and immunotherapy.^5^ The retrospective AVATAX study found that bevacizumab plus paclitaxel yielded longer median PFS in ICI‐pretreated patients than in ICI‐naïve patients, with median PFS of 7.0 versus 5.7 months (*p* = 0.01).^13^ This evidence suggests that antiangiogenic agents may achieve greater efficacy in ICI‐treated patients, providing a strong rationale for incorporating antiangiogenic agents after failure of immunotherapy.

Another strategy is platinum re‐challenge. Although platinum‐free interval (PFI) is a known predictor of platinum sensitivity in ovarian and small cell lung cancer, its role in NSCLC remains controversial. One large retrospective analysis found no survival benefit from platinum re‐challenge in ICI‐pretreated NSCLC, irrespective of PFI length, although nsqNSCLC‐specific data were not provided.^14^ Another real‐world retrospective study reported that platinum re‐challenge combined with antiangiogenic therapy yielded the longest PFS and OS among several second‐line regimens, yet again nsqNSCLC subgroup analysis was lacking.^15^ Similarly, a retrospective study in patients with unresectable NSCLC who failed radical chemoradiotherapy showed significant improvement in OS with platinum re‐challenge compared with the non‐platinum re‐challenge subgroup.^16^ Interestingly, greater survival benefit from platinum re‐challenge was observed in patients whose PFS of radical chemoradiotherapy was shorter than the median.^16^ As longer PFS typically corresponds to a longer PFI, the result indicates that PFI length is not a reliable biomarker for platinum re‐challenge. A prospective single‐arm study is currently underway to evaluate the efficacy and safety of platinum re‐challenge in advanced or metastatic NSCLC.^17^ As first‐line immunochemotherapy significantly prolongs survival and lengthens PFI, potentially restoring platinum sensitivity, platinum re‐challenge may be a viable option.

In the pre‐immunotherapy era, bevacizumab combined with paclitaxel and platinum was a standard first‐line regimen for advanced nsqNSCLC.^18^ Nowadays, most patients receive first‐line ICIs in combination with pemetrexed and platinum and are naïve to bevacizumab and paclitaxel. Introducing the triplet in second line may be a plausible salvage option. A small retrospective study of 30 patients with advanced NSCLC reported median PFS and OS of 6.3 and 11.8 months and an ORR of 66.7% with bevacizumab‐paclitaxel‐carboplatin in late‐line settings, though the inclusion of epidermal growth factor receptor (EGFR)‐mutant and ICI‐naïve patients confounded interpretation.^19^

Based on these considerations, we conducted a prospective single‐arm, single‐centre study to evaluate the efficacy and safety of bevacizumab combined with nab‐paclitaxel and platinum (the BAP regimen) as a second‐line therapy for driver‐gene‐negative ICI‐treated nsqNSCLC. This regimen aims to leverage the synergistic potential of antiangiogenic therapy, cytotoxic therapy and platinum re‐challenge, utilising widely available agents to improve survival outcomes.

## 2 METHODS

### 2.1 Study design and participants

This was a single‐centre, single‐arm prospective clinical trial designed to evaluate the efficacy and safety of bevacizumab plus nab‐paclitaxel and platinum (the BAP regimen) as second‐line therapy in patients with driver‐gene‐negative advanced nsqNSCLC who progressed after first‐line immunotherapy (BETTER trial, ClinicalTrials.gov identifier: NCT05407155). Key eligibility criteria included: Age 18–75 years; histologically or cytologically confirmed unresectable, locally advanced or metastatic, stage IIIB–IV nsqNSCLC (AJCC 8th edition), not suitable for curative treatment; absence of EGFR mutations, ALK rearrangements, or ROS1 rearrangements; progressive disease after first‐line ICI‐based therapy, including PD‐1, PD‐L1 inhibitors with/without CTLA‐4 inhibitors; at least one measurable lesion per Response Evaluation Criteria in Solid Tumours (RECIST) v1.1; central nervous system metastases were allowed, as long as physicians decided that patients were suitable for the BAP regimen. Key exclusion criteria included: mixed histology including small cell and squamous lung cancer; prior treatment with antiangiogenic agents or any paclitaxel formulation in the advanced setting; patients who had received paclitaxel in the adjuvant setting were eligible if recurrence occurred more than 6 months after completion; uncontrolled hypertension, significant cardiovascular disease, active infections or other serious comorbidities; clinically significant haemoptysis, thrombosis or bleeding disorders. No randomisation or blinding was needed.

### 2.2 Treatment

Eligible patients received the BAP regimen, which included intravenous bevacizumab 15 mg/kg plus nab‐paclitaxel 260 mg/m^2^ and platinum (carboplatin area under the curve = 5 or cisplatin 75 mg/m^2^, chosen by physicians) on day 1 every 3 weeks for 4 to 6 cycles, followed by maintenance therapy of intravenous bevacizumab 15 mg/kg on day 1 every 3 weeks until disease progression or unacceptable toxicity. Dose adjustments were permitted for heamatologic and non‐hematologic toxicities according to predefined criteria for nab‐paclitaxel and platinum. Bevacizumab dose reductions were not permitted, but it could be withheld or discontinued for related adverse events (AEs).

### 2.3 Endpoints

The primary endpoint was ORR, defined as the proportion of patients achieving a complete response (CR) or partial response (PR) according to RECIST v1.1. All objective responses needed to be confirmed. All imaging evaluations were conducted by the investigators. Secondary endpoints included PFS, OS, disease control rate (DCR), depth of response (DpR) and safety. Tumour imaging was performed with computed tomography (CT) or magnetic resonance imaging at screening, every two treatment cycles until week 54, after which every three treatment cycles until disease progression, or every 9 weeks for patients who discontinued treatment, or at any time deemed clinically necessary. PR and CR were confirmed at the next tumour imaging assessment (usually conducted more than 4 weeks later). Patients without evaluable post‐baseline assessments for any reason were classified as not evaluable (NE). PFS was defined as the time from informed consent to disease progression or death from any cause, whichever comes first. OS was defined as the time from informed consent to death from any cause. DCR was defined as the proportion of patients achieving a CR, PR or SD according to RECIST v1.1. DpR was defined as the maximum percentage change in the sum of longest diameters of RECIST target lesions at the nadir compared to baseline, with NE patients excluded from the analysis. Incidence and severity of AEs were graded according to NCI‐CTCAE v5.0. AEs were monitored throughout the study until 30 days after the last dose of study drug. Exploratory subgroup analyses were performed based on clinicopathological characteristics and plasma proteins to identify potential biomarkers.

### 2.4 Proteomic analysis

Peripheral blood samples prior to treatment of 47 patients were collected after informed consent for biomarker analysis. Plasma was separated from blood and preserved at ‐80°C. Among the 47 patients, 31 patients had previously been enrolled in a clinical trial in Fudan University Shanghai Cancer Centre (FUSCC) for first‐line immunotherapy (MIRNA‐SEP‐01, NCT04427475), and preserved plasma samples were available for further study. Plasma samples were tested using the Olink Target 96 Immune‐Oncology panel (Catalogue No. 95311) to explore potential proteomic biomarkers. Procedures were as follows. Proximity Extension Assay on the Olink platform uses a matched pair of antibodies linked to unique oligonucleotides that bind to the target protein. The resulting DNA amplicon was quantified by quantitative real‐time polymerase chain reaction (qRT‐PCR). During incubation, 92 antibody pairs labelled with DNA oligonucleotides bound to their target proteins in the samples. During extension and amplification, the oligonucleotides were brought into proximity via hybridisation and were extended by a DNA polymerase. The DNA barcodes were subsequently amplified by PCR. The microfluidic qRT‐PCR output was quantified with the Olink Signature Q100 instrument, and NPX values were generated using the Olink NPX Signature software. Incubation, extension, and detection steps were performed by Sinotech Genomics Co., Ltd. (Shanghai, China). NPX values were calculated from threshold cycles, and data pre‐processing (normalisation) was applied to minimise intra‐ and inter‐assay variation. Protein levels for each target across samples were derived from NPX values.

### 2.5 Statistical analysis

The sample size was determined under the assumption that the experimental regimen would increase the ORR from 15%^20^, ^21^, ^22^, ^23^ (historical data of docetaxel treatment) to 30%, with a two‐sided significance level of 0.05, 80% detection power, and an anticipated dropout rate of 5%. A sample size of 56 patients was planned. The 95% confidence interval (CI) of ORR was calculated using the Wilson procedure without a continuity correction. The statistical significance of the difference between observed ORR and historical null rate of 15% was calculated using an Exact binomial test. PFS and OS were estimated using the Kaplan‐Meier method in the Intent‐to‐Treat (ITT) population, which was defined as all enrolled patients regardless of adherence or withdrawal.^24^ Safety data were summarised descriptively in the Safety population, defined as the population who received at least one dose of study treatment. Categorical variables were compared using the chi‐square test. For multiple comparisons, the Benjamini‐Hochberg procedure was used to control the false discovery rate (FDR), with FDR set at 0.1. Survival comparisons were performed using Log‐rank tests in the Per‐Protocol (PP) population, defined as patients who adhered to the planned treatment, with the exclusion of the four patients who withdrew prior to treatment initiation.^24^ Multivariable analysis was performed using Cox proportional hazards regression. Differential protein expression was analysed using the Olink Analyze R package (version 3.1.0). Continuous variables were compared using Student's *t*‐test. All statistical tests were two‐tailed, and a *p*‐value of less than 0.05 was considered statistically significant.

## 3 RESULTS

### 3.1 Participants

Between 9 June 2022 and 31 July 2025, a total of 56 patients with advanced driver‐gene‐negative nsqNSCLC who had progressed after first‐line immunochemotherapy were enrolled. Baseline characteristics are summarised in Table 1. All patients had received first‐line ICI plus pemetrexed and platinum (100%), with the most common ICI agent being pembrolizumab (42.9%), followed by tislelizumab (32.1%). The median age was 60 (range, 28–75) years, and the majority were male (76.8%). The most common metastatic organs were lung, lymph nodes, bone and pleura. Notably, 15 patients (26.8%) had central nervous system metastasis, including one patient with cytologically confirmed meningeal metastasis. Thirty‐six patients (64.3%) had three or more metastatic organs, indicating a high metastatic burden. PD‐L1 expression was unknown in half of the patients (50.0%). A total of 52 patients received at least one dose of study treatment, and the remaining four patients refused per‐protocol treatment and were censored.

**TABLE 1:** Patient characteristics at baseline.

Characteristic	Number of patients (%) (*N* = 56)
Median age (years, range)	60 (28–75)
Smoking status	
Previous or current	31 (55.4%)
Never	22 (39.3%)
Unknown	3 (5.4%)
Sex	
Male	43 (76.8%)
Female	13 (23.2%)
Metastatic sites	
Lung	41 (73.2%)
Regional lymph nodes	35 (62.5%)
Bone	24 (42.9%)
Pleura	17 (30.4%)
Central nervous system	15 (26.8%)
Brain	14 (25.0%)
Meninges	1 (1.8%)
Liver	10 (17.9%)
Adrenal glands	9 (16.1%)
Distant lymph nodes	4 (7.1%)
Others	14 (25.0%)
Number of metastatic organs	
1	10 (17.9%)
2	10 (17.9%)
≥3	36 (64.3%)
PD‐L1	
< 1%	14 (25.0%)
1%–49%	10 (17.9%)
≥50%	4 (7.1%)
Unknown	28 (50.0%)
Previous treatment	
Chemo‐free Immunotherapy	0 (0%)
Immunotherapy plus chemotherapy	56 (100%)
Pembrolizumab + pemetrexed + platinum	24 (42.9%)
Tislelizumab + pemetrexed + platinum	18 (32.1%)
Camrelizumab + pemetrexed + platinum	6 (10.7%)
Other ICIs + pemetrexed + platinum	8 (14.3%)
Current treatment	
Bevacizumab + nab‐paclitaxel + carboplatin	49 (87.5%)
Bevacizumab + nab‐paclitaxel + cisplatin	3 (5.4%)
Refusal of per‐protocol treatment	4 (7.1%)

Abbreviation: ICI, immune checkpoint inhibitor.

### 3.2 Efficacy

**FIGURE 1:**
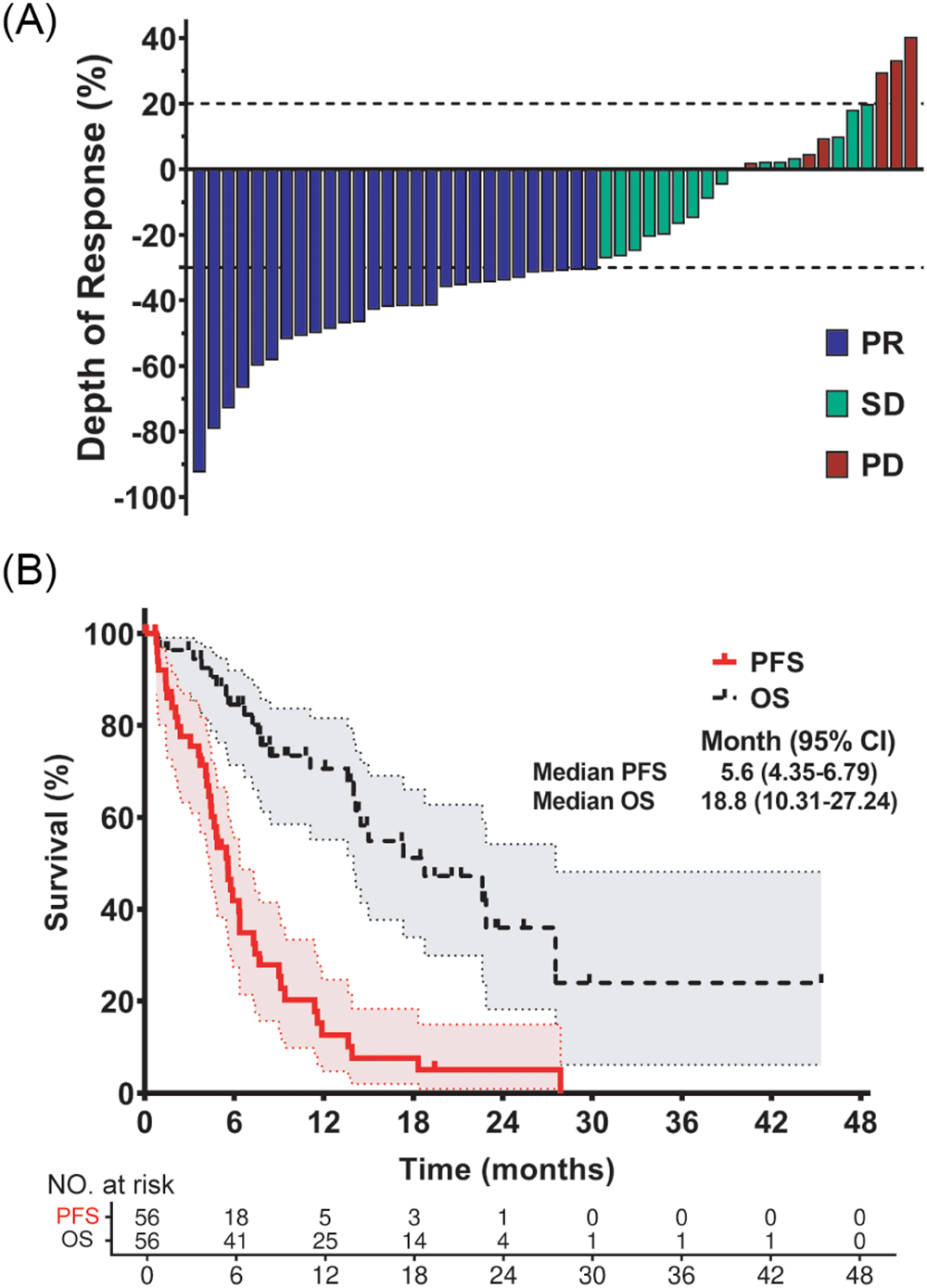
Efficacy for patients receiving second‐line bevacizumab plus nab‐paclitaxel and platinum therapy. (A) The waterfall plot of depth of response of patients treated with the second‐line BAP regimen. Patients without evaluable post‐baseline imaging (*n* = 6) were excluded. Bars are colour‐coded according to best overall response (PR, blue; SD, green; PD, red). The lower dashed line indicates the cutoff for PR (−30%), and the upper dashed line indicates the cutoff for PD (+20%). (B) Kaplan‐Meier curves of progression‐free survival (PFS) and overall survival (OS) of patients treated with the second‐line BAP regimen.

At the time of the data cutoff date of 15 April 2026, median follow‐up time was 17.2 months. In the ITT population, the ORR was 50.0% (28/56), all of which were PRs (Table 2). Stable disease was reported in 14 patients (Table 2, 25.0%). DCR was 75% (Table 2). Six patients (10.7%) were NE for response, including four patients refusing per‐protocol treatment, one patient lost to follow‐up, and one patient who died due to AE before any response evaluation (Table 2). The confirmed ORR was 46.4% (Table 2, 26/56, 95% CI: 34.0%–59.3%), which was significantly higher than the historical control rate of 15% (exact binomial test, *p* < 0.001). A waterfall plot of DpR in evaluable patients was shown in Figure 1A. A total of 37 out of 56 (66.1%) patients achieved tumour shrinkage from baseline. The observed ORR demonstrated a meaningful clinical benefit over current second‐line options. Among patients enrolled, 15 had central nervous system metastases. Four patients received intracranial radiotherapy during first‐line treatment, and one received intracranial radiotherapy 2 weeks before current treatment. Of the 15 patients, 10 had measurable intracranial lesions, and the intracranial ORR was 30% (3/10), all of which were PR, and DCR was 50% (Table S1). At the time of the data cutoff date, median PFS was 5.6 (95% CI: 4.35–6.79) months, and median OS was 18.8 (95% CI: 10.31–27.24) months in the ITT group (Figure 1B).

**TABLE 2:** Objective response.

Response	Number of patients (%) (*N* = 56)
Complete response	0 (0%)
Partial response	28 (50.0%)
Stable disease	14 (25.0%)
Progressive disease	8 (14.3%)
Not evaluatable	6 (10.7%)
ORR	28 (50.0%)
DCR	42 (75.0%)
Confirmed ORR	26 (46.4%)

Abbreviations: DCR, disease control rate; ORR, objective response rate.

**FIGURE 2:**
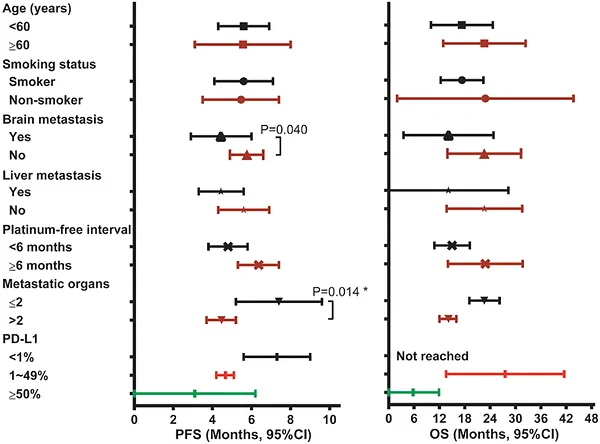
Forest plots of progression‐free survival (PFS) and overall survival (OS) (median, 95% confidence interval [CI]) by different baseline characteristics. *: The *p*‐values were significant after being adjusted for multiple testing using the Benjamini‐Hochberg method, with a false discovery rate <0.1.

Further exploratory subgroup analysis was performed in the PP population, since four patients refused per‐protocol treatment. Patients with confirmed PR or CR were classified as responders, while others with PD, SD, unconfirmed PR or CR, and NE were classified as non‐responders in our study. Pearson's chi‐squared tests showed that liver metastasis, non‐response to first‐line immunochemotherapy, and PFI <6 months were significantly correlated with non‐response to the BAP treatment (*p* = 0.021, *p* = 0.035 and *p* = 0.006, respectively), while central nervous system metastasis was not correlated with response (Table S2). Log‐Rank tests showed that patients with ≤2 metastatic organs had significantly longer median PFS than those with >2 metastatic organs (*p* = 0.014, Figure 2), and patients without brain metastases also had significantly longer median PFS than those with brain metastases (*p* = 0.04; Figure 2). After adjusting the above *p*‐values for multiple testing using the Benjamini‐Hochberg method with FDR < 0.1, only metastatic organs ≤2 remained a significant predictor of prolonged PFS (Figure 2). Liver metastasis was not correlated with PFS and OS (Figure 2). Cox multivariable analysis showed that only PFI was an independent biomarker for PFS (PFI ≥ 6 vs. < 6 months: *p* = 0.038, hazard ratio 0.49, Table 3).

**TABLE 3:** Cox multivariable analysis of factors associated with progression‐free survival and overall survival.

	Progression‐free survival			Overall survival		
Characteristics (*N* = 52)	*p*‐Value	HR	95%CI	*p*‐Value	HR	95%CI
Age group						
≤60	Ref					
>60	0.937	1.03	0.53–2.01	0.760	0.87	0.35–2.18
Smoking						
No	Ref					
Previous or current smoker	0.787	0.90	0.42–1.93	0.598	0.76	0.27–2.12
Brain metastasis						
No	Ref					
Yes	0.317	1.56	0.65–3.74	0.250	1.96	0.62–6.13
Liver metastasis						
No	Ref					
Yes	0.759	1.17	0.43–3.21	0.701	1.30	0.34–5.05
Number of metastatic organs						
≤2	Ref					
>2	0.090	1.90	0.90–4.00	0.871	1.09	0.38–3.15
Platinum‐free interval						
<6 months	Ref					
≥6 months	**0.038**	**0.49**	0.25–0.96	0.163	0.51	0.20–1.31

### 3.3 Safety

A total of 52 patients who received at least one cycle of study treatment were included in the safety analysis. Treatment‐related AEs occurred in 52 (100%) patients. The most common any‐grade AEs included alopecia, fatigue, hypertension, neutropenia and anaemia (Table 4). Severe AEs (Grade 3 or higher) occurred in 15 (28.8%) patients, with the most common severe AEs being neutropenia, fatigue and leukopenia. Grade 4 anaphylaxis to carboplatin occurred in two patients during cycles 1 and 5, respectively. The patient who experienced Grade 4 carboplatin‐induced anaphylaxis in cycle 1 subsequently dropped out of the study. Both patients permanently discontinued carboplatin treatment. Grade 5 bleeding event of haemoptysis occurred in one patient (1.9%), which led to the patient's immediate death 1 week after Cycle 2 of the BAP treatment. Response evaluation could not be performed for this patient. No new safety signals were identified.

**TABLE 4:** Adverse events.

Adverse events (*N* = 52)	Grades 1 and 2	Grades 3–5	Total
	*N* (%)	*N* (%)	*N* (%)
All adverse events	52 (100%)	15 (28.8%)	–
Alopecia	52 (100%)	–	52 (100%)
Fatigue	15 (28.8%)	8 (15.4%)	23 (44.2%)
Hypertension	17 (32.7%)	3 (5.8%)	20 (38.5%)
Neutropenia	4 (7.7%)	11 (21.2%)	15 (28.8%)
Anaemia	14 (26.9%)	1 (1.9%)	15 (28.8%)
Leukopenia	5 (9.6%)	8 (15.4%)	13 (25.0%)
Anorexia	9 (17.3%)	4 (7.7%)	13 (25.0%)
Peripheral sensory neuropathy	6 (11.5%)	5 (9.6%)	11 (21.2%)
Increased ALT/AST	8 (15.4%)	0	8 (15.4%)
Thrombocytopenia	4 (7.7%)	3 (5.8%)	7 (13.5%)
Rash	7 (13.5%)	0	7 (13.5%)
Proteinuria	5 (9.6%)	2 (3.8%)	7 (13.5%)
Vomiting	5 (9.6%)	1 (1.9%)	6 (11.5%)
Hypercalcemia	3 (5.8%)	0	3 (5.8%)
Constipation	3 (5.8%)	0	3 (5.8%)
Hypokalemia	2 (3.8%)	0	2 (3.8%)
Anaphylaxis	0	2 (3.8%)	2 (3.8%)
Fever	2 (3.8%)	0	2 (3.8%)
Weight loss	1 (1.9%)	1 (1.9%)	2 (3.8%)
Bleeding events	1 (1.9%)	1 (1.9%)	2 (3.8%)

Abbreviations: ALT, alanine aminotransferase; AST, aspartate aminotransferase.

### 3.4 Biomarker exploration

**FIGURE 3:**
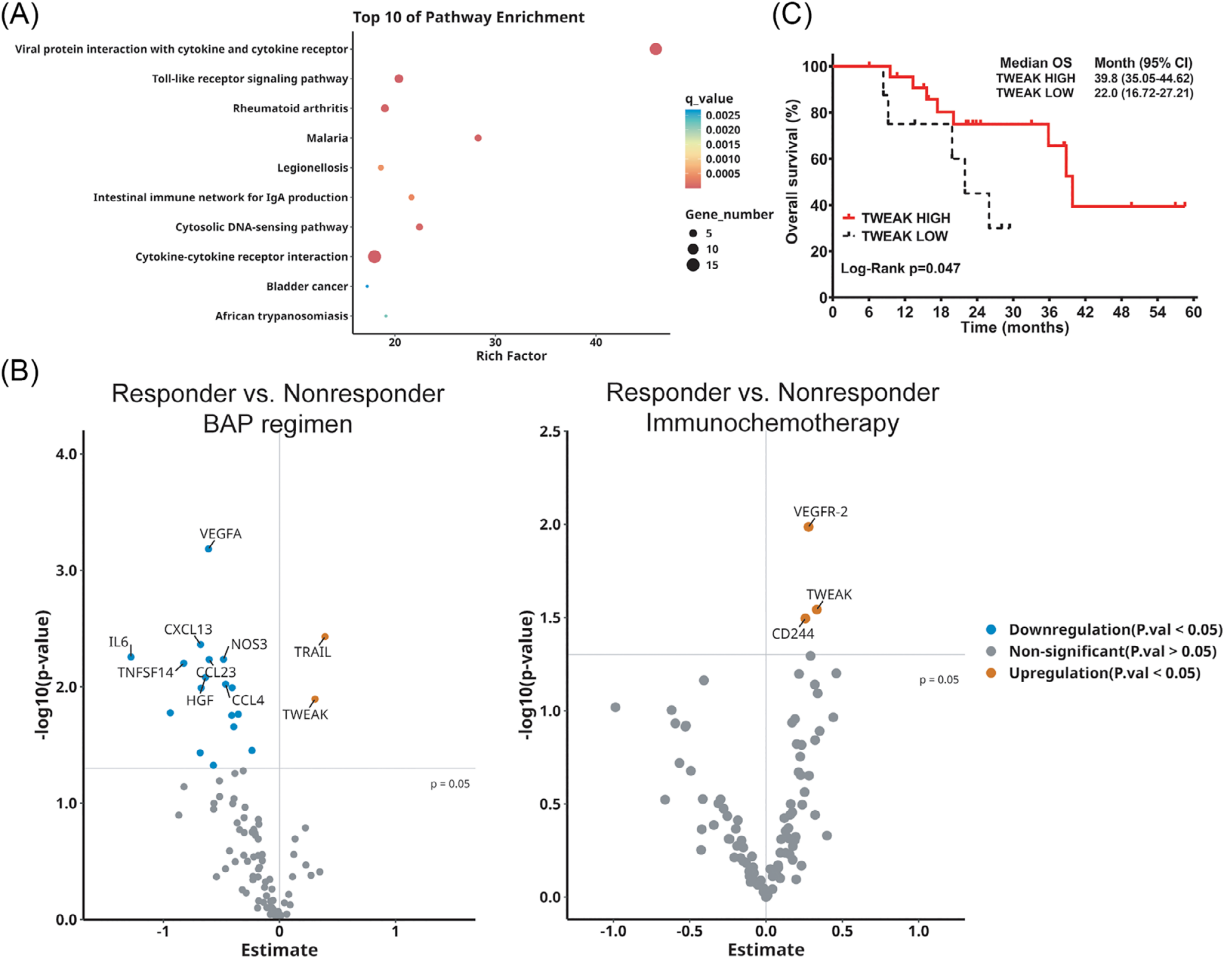
Identification of differential plasma proteins as biomarkers for efficacy. (A) KEGG pathway analysis of differential plasma proteins between responders and non‐responders to the BAP regimen. Differential proteins were enriched in pathways related to immune responses and cytokines. (B) Volcano plots showed differential plasma proteins between responders and non‐responders to the BAP regimen (left) and to immunochemotherapy (right). (C) Kaplan‐Meier curves of overall survival (OS) in patients with high and low baseline plasma TWEAK levels before first‐line immunochemotherapy.

A plasma proteomic analysis was performed using the Olink Target 96 Immuno‐Oncology panel to detect potential biomarkers associated with efficacy. KEGG pathway analysis of differential proteins between responders (n = 24) and non‐responders (*n* = 23) to the BAP regimen showed enrichment of proteins in pathways related to immune responses and cytokines (Figure 3A). Differential plasma proteins were shown in Figure 3B. Notably, plasma VEGFA level was significantly lower in responders than in non‐responders to the BAP regimen (Figure 3B). Plasma samples collected prior to initiation of first‐line immunochemotherapy treatment from 31 patients in our cohort were available for plasma proteomic analysis. Results showed that compared to non‐responders (*n* = 13) to first‐line immunochemotherapy, baseline plasma levels of VEGFR2, TWEAK and CD244 in responders (*n* = 18) were significantly higher (Figure 3B). Unexpectedly, as angiogenesis is a well‐known resistance pathway to immunotherapy, high plasma levels of VEGFR2 were correlated with better response to immunochemotherapy in our cohort.

Interestingly, plasma levels of TWEAK, also known as TNFSF12, were a biomarker of response to both first‐line immunochemotherapy and second‐line BAP therapy (Figure 3B). TWEAK is a member of the TNF superfamily, which modulates multiple cellular responses including angiogenesis and production of proinflammatory mediators. However, the clinical significance and biological roles of TWEAK remain largely unknown. We defined the cut‐off values for TWEAK according to the receiver operating characteristic curve for responses (Figure S1A,B). Survival analysis showed that patients with higher baseline plasma TWEAK levels before first‐ or second‐line treatment had a tendency towards longer median PFS for each line, respectively (Figure S1C,D). Patients with higher TWEAK plasma levels before immunochemotherapy had significantly longer median OS from the beginning of first‐line therapy (39.8 vs. 22.0 months, *p* = 0.047, Figure 3C). Our exploratory findings indicated that baseline TWEAK prior to first‐line treatment may be a promising biomarker for driver‐gene‐negative advanced nsqNSCLC under our sequential therapeutic modality, and suggested the value of monitoring the dynamic changes of TWEAK.

## 4 DISCUSSION

Treatment after progression on immunotherapy remains challenging for advanced driver‐gene‐negative NSCLC. Current standard second‐line therapies remain docetaxel monotherapy or its combination with antiangiogenic agents (such as ramucirumab or nintedanib).^20^, ^25^, ^26^ Earlier trials, such as REVEL and LUME‐Lung 1, enrolled ICI‐naïve patients and showed that docetaxel plus ramucirumab or nintedanib moderately improved PFS and OS to approximately 4.5 and 12 months, respectively, versus docetaxel monotherapy.^20^, ^26^ In the immunotherapy era, the VARGADO trial reported a median PFS of 4.8 months and an ORR of 37.5% with docetaxel plus nintedanib in ICI‐pretreated patients.^25^ However, efficacy remains limited.

Numerous post‐immunotherapy strategies are under active investigation for driver‐gene‐negative nsqNSCLC. Chemo‐free regimens such as continuing ICIs treatment beyond progression in combination with targeted drugs have shown preliminary activity. In the HUDSON trial, a biomarker‐directed umbrella study, durvalumab plus ceralasertib significantly improved outcomes in ICI‐treated NSCLC patients carrying *ATM* alterations, particularly in the nsqNSCLC subgroup, of whom median PFS and OS reached 6.0 and 18.8 months, respectively.^6^ In the Lung‐MAP S1800A trial, pembrolizumab plus ramucirumab improved OS versus physicians’ choice (14.5 vs. 11.6 months) in patients failing immunochemotherapy, whereas PFS did not differ between arms.^7^ Both regimens are currently under further evaluation in large trials. However, other combinations of cross‐line PD‐1/PD‐L1 antibodies with tyrosine kinase inhibitors failed to demonstrate significant survival benefits, and the ORRs remained at approximately 20%, suggesting limited tumour remission with a chemo‐free regimen.^8^, ^9^, ^10^

ADCs represent a novel class of therapeutics that combine targeted drug delivery with cytotoxic payloads. However, attempts to establish an ADC‐based post‐ICI treatment strategy have encountered setbacks. TROPION‐Lung01 trial demonstrated a statistically significant improvement in PFS and ORR achieved by datopotamab deruxtecan, a TROP‐2‐directed ADC, compared to docetaxel for nsqNSCLC, but failed to demonstrate a significant improvement in OS.^12^ Another TROP‐2‐directed ADC, sacituzumab govitecan, failed to demonstrate significant improvements in PFS, OS or ORR compared to docetaxel in the EVOKE‐01 trial.^11^ A trial of a CEACAM5‐directed ADC was halted due to lack of significant improvement in median PFS.^27^ Other modalities, including ADCs for other targets, bispecific antibodies and cancer vaccines, remain in early development.^5^ Therefore, options for post‐ICI salvage therapy remain limited.

To our knowledge, this study is the first prospective study to evaluate the efficacy and safety of bevacizumab plus nab‐paclitaxel and platinum triplet (the BAP regimen) as second‐line therapy in advanced driver‐gene‐negative nsqNSCLC failing immunochemotherapy. In this study, the BAP regimen yielded a confirmed ORR of 46.4%, a median PFS of 5.6 months, and a median OS of 18.8 months, with a manageable safety profile. The regimen integrates the synergistic potential of antiangiogenic therapy, the cytotoxic effect, and platinum re‐challenge. Additionally, another advantage of the BAP regimen is the combination of highly accessible drugs, providing a potential option for salvage treatment after failing immunotherapy. Although similar post‐ICI treatment strategies have been reported as effective in the AVATAX trial and a small sample study, both had a retrospective nature and confounding factors. Given that all patients enrolled in our study were platinum‐treated, the efficacy and survival outcomes observed with the BAP regimen suggest preliminary clinical activity in this pretreated population, which warrants evaluation in randomised controlled trials. Multivariable analysis found that PFI was an independent factor correlated with PFS. Neither brain metastasis, liver metastasis, nor number of metastatic organs was significantly correlated with survival. PD‐L1 expression was unknown in half of the patients in our study, which is a limitation. As first‐line immunochemotherapy for driver‐gene‐negative nsqNSCLC can be administered regardless of PD‐L1 status in China, PD‐L1 testing was not mandatory in some centres. We believe this reflects real‐world clinical practice, but should be addressed in future studies.

In biomarker analysis, comparison of plasma proteomics between responders and non‐responders to the BAP regimen revealed significantly enriched pathways related to immune responses and cytokines. Low baseline plasma level of VEGFA was a potential biomarker for response to the BAP regimen. Plasma VEGFA levels are commonly incorporated into biomarker analysis of anti‐angiogenic therapies. Our finding was consistent with the recent biomarker analysis of the APPLE trial.^28^ Although the APPLE trial did not meet its primary endpoint of demonstrating the superiority of adding bevacizumab to platinum‐doublet plus the PD‐L1 inhibitor atezolizumab in metastatic or recurrent nsqNSCLC, its biomarker analysis showed that in the EGFR wild‐type subgroup, low serum VEGFA was significantly correlated with greater benefit from the addition of bevacizumab to immunotherapy.^28^

We further assessed the predictive value of plasma proteins in first‐line immunochemotherapy. Angiogenesis is a well‐known resistance mechanism to immunotherapy, but we did not observe a predictive effect of plasma VEGFA for immunochemotherapy. However, we found that high plasma levels of VEGFR2, the receptor of VEGFA, were associated with a better response to immunochemotherapy. The role of VEGFR2 as a predictive biomarker for the efficacy of immunotherapy remains unclear. A small‐sized study reported that high expression of VEGFR2 in tumours correlated with an immunosuppressive tumour environment and worse prognosis in NSCLC treated with PD‐1 antibody, which was contradictory to our finding.^29^ However, the study did not assess peripheral VEGFR2 levels. In addition, we should not forget that a single biomarker cannot represent the full landscape of angiogenesis, and further investigations are needed.

It is noteworthy that we identified significantly higher plasma levels of TWEAK/TNFSF12 in both responders to first‐line immunochemotherapy and second‐line BAP therapy compared to non‐responders. As a member of the TNF superfamily, TWEAK modulates multiple cellular responses, including angiogenesis and inflammation. TWEAK has dual roles in different tumour microenvironments. On one hand, in gastric cancer, plasma TWEAK levels were significantly upregulated in poor responders to regorafenib plus avelumab treatment, possibly through M2 macrophage recruitment and polarisation.^30^ On the other hand, TWEAK expression in breast cancer tissues was positively correlated with infiltration of memory B cells and CD8^+^ T cells, and may play a protective role.^31^ In our study, higher plasma TWEAK level before each line of treatment showed a trend toward longer PFS, respectively, although this did not reach statistical significance, partially due to small sample size. We also found that higher plasma TWEAK level before first‐line immunochemotherapy was significantly correlated with longer OS in our cohort, with the TWEAK‐high group achieving median OS of 39.8 months from the beginning of first‐line therapy. While the observed survival time was numerically longer than those reported in some clinical trials of similar settings, cross‐trial comparison is not appropriate, and our results should be interpreted with caution. However, it should not be overlooked that patients enrolled in our study were more likely to tolerate a platinum‐based triplet therapy, indicating a better performance status than other real‐world patients, which may confound the observed longer OS. Therefore, our results should be interpreted with caution. Nevertheless, our results indicated that plasma TWEAK may serve as a potential stratification biomarker to identify patients who would benefit most from this sequential treatment modality, pending independent validation. However, the functioning mechanisms of TWEAK in lung cancer remain unclear. Whether TWEAK represents the interplay between immune response and angiogenesis warrants further investigation.

The toxicity of the study regimen was manageable, without new safety signals identified. But we did observe Grade 4 anaphylaxis to carboplatin in two patients and a Grade 5 bleeding event of haemoptysis in 1 patient, underscoring the need for close monitoring and careful exclusion of tumours invading large vessels.

In summary, this single‐arm, single‐centre, prospective study provides preliminary evidence of the efficacy and safety of bevacizumab plus nab‐paclitaxel and platinum as a second‐line treatment after failure of immunochemotherapy for driver‐gene‐negative advanced nsqNSCLC. Dynamic plasma TWEAK levels may serve as a potential biomarker to identify patients most likely to benefit from this sequential treatment modality. However, our study was limited by a small sample size and lack of a randomised control group. Future prospective and randomised studies are needed to further evaluate the efficacy of the BAP regimen and the predictive role of TWEAK.

## AUTHOR CONTRIBUTIONS

Conceptualization: Jialei Wang; Investigation: Ying Lin, Zhihuang Hu, Yao Zhang, Zhenhua Wu, Bo Yu, Xinmin Zhao, Hui Yu, Xianghua Wu, Huijie Wang and Jialei Wang; Performing clinical trial: Ying Lin, Zhihuang Hu, Chenchen Wei, Yao Zhang, Zhenhua Wu, Bo Yu, Xinmin Zhao, Hui Yu, Xianghua Wu, Huijie Wang and Jialei Wang; Original draft preparation: Ying Lin, Zhihuang Hu, Chenchen Wei and Yunbo Yu; Writing review & editing: Ying Lin, Zhihuang Hu, Chenchen Wei, Yunbo Yu, Yao Zhang, Zhenhua Wu, Bo Yu, Xinmin Zhao, Hui Yu, Xianghua Wu, Huijie Wang and Jialei Wang; Data analysis: Ying Lin, Zhihuang Hu, Chenchen Wei and Yunbo Yu; Patient recruitment: Ying Lin, Zhihuang Hu, Yao Zhang, Zhenhua Wu, Bo Yu, Xinmin Zhao, Hui Yu, Xianghua Wu, Huijie Wang and Jialei Wang; Supervision: Jialei Wang.

## CONFLICT OF INTEREST STATEMENT

The authors declare no conflict of interest.

## FUNDING INFORMATION

This study was supported by the Collaborative Innovation Cluster Project of the Shanghai Municipal Commission of Health and Family Planning (2020CXJQ02), Shanghai's 2022 “Science and Technology Innovation Action Plan” Special Project in Medical Innovation Research (22Y31920405) and Beijing Life Oasis Public Welfare Service Centre (CPHCF‐ZLKY‐2023016).

## ETHICS STATEMENT

This study was approved by FUSCC Ethics Committee (approval number: 2201250‐11), and performed in accordance with the principles in the 1964 Helsinki Declaration.

## PATIENT CONSENT STATEMENT

Informed consent was obtained from every patient in accordance with the guidelines of the FUSCC Ethics Committee.

## CLINICAL TRIAL REGISTRATION

ClinicalTrials.gov identifier: NCT05407155.

## Supporting information




**FIGURE S1** Identification of baseline plasma TWEAK protein level as a biomarker. (A, B) Receiver operating characteristic (ROC) curve of baseline plasma TWEAK as a biomarker of the second‐line BAP regimen (A) and first‐line immunochemotherapy (B). (C, D) Kaplan‐Meier curves of PFS according to baseline plasma TWEAK levels before second‐line BAP treatment (C) and before first‐line immunochemotherapy (D).

Supporting Information

## Data Availability

The data that support the findings of this study are available on reasonable request from the corresponding author without compromising patients’ privacy.
