# Supplementary material for: Bevacizumab plus nab‐paclitaxel and platinum as second‐line therapy for driver‐gene‐negative non‐squamous non‐small cell lung cancer after immunochemotherapy: A prospective single‐arm single‐centre study
Source: Clin Transl Med. 2026 Sep 11;16(9):e70814. doi: 10.1002/ctm2.70814 (PMC13563325; doi:10.1002/ctm2.70814)
Supplement: Supplementary file 1 [file CTM2-16-e70814-s002.docx]

**Supplementary Table 1.** Intracranial objective response

| Intracranial Response | Number of patients (%) |
| --- | --- |
| With measurable lesions | N=10 |
| Complete response | 0 (0%) |
| Partial response | 3 (30.0%) |
| Stable disease | 2 (20.0%) |
| Progressive disease | 3 (30.0%) |
| Not evaluated | 2 (20.0%) |
| ORR | 3 (30.0%) |
| DCR | 5 (50.0%) |
| Without measurable lesions | N=5 |
| Non-CR/non-PD | 4 (80%) |
| Progressive disease | 1 (20.0%) |

Abbreviations: ORR: objective response rate; DCR: disease control rate

**Supplementary Table 2.** Characteristics correlated with response

| Characteristics | Responders  (N=26) | Non-responders  (N=26) | P value |
| --- | --- | --- | --- |
| Age |  |  |  |
| <60 | 12 | 12 | 1.000 |
| ≥60 | 14 | 14 |  |
| Smoking status |  |  |  |
| Previous or current | 14 | 16 | 0.771 |
| Never | 10 | 9 |  |
| Unknown | 2 | 1 |  |
| Sex |  |  |  |
| Male | 18 | 23 | 0.090 |
| Female | 8 | 3 |  |
| Central nervous system metastasis |  |  |  |
| Yes | 7 | 7 | 1.000 |
| No | 19 | 19 |  |
| Liver |  |  |  |
| Yes | 1 | 7 | **0.021** |
| No | 25 | 19 |  |
| Number of metastatic organs |  |  |  |
| ≤2 | 11 | 8 | 0.388 |
| >2 | 15 | 18 |  |
| Response to first-line immunotherapy |  |  |  |
| Responder | 24 | 18 | **0.035** |
| Non-responder | 2 | 8 |  |
| Platinum-free interval |  |  |  |
| <6 months | 8 | 18 | **0.006** |
| ≥6 months | 18 | 8 |  |
| PD-L1 |  |  |  |
| <1% | 8 | 5 | 0.545 |
| 1~49% | 4 | 6 |  |
| ≥50% | 1 | 3 |  |
| Unknown | 13 | 12 |  |
